# Optimal sequence of LT for symptomatic BM in EGFR-mutant NSCLC: a comparative study of first-line EGFR-TKIs with/without upfront LT

**DOI:** 10.1007/s00432-023-05538-9

**Published:** 2024-02-19

**Authors:** Lishui Niu, Honghua Wu, Ruihuan Gao, Liu Chen, Jiangtao Wang, Hexin Duan, Yujiao Long, Yi Xie, Qin Zhou, Rongrong Zhou

**Affiliations:** 1 Department of Oncology, Xiangya Hospital, Central South University, 87 Xiangya Road, Kaifu District, Changsha, 410008 Hunan China; 2 Department of Oncology, Xiangxi Autonomous Prefecture People’s Hospital, Jishou, 416000 China; 3 Xiangya Lung Cancer Center, Xiangya Hospital, Central South University, Changsha, 410008 China; 4 National Clinical Research Center for Geriatric Disorders, Xiangya Hospital, Central South University, Changsha, 410008 China

**Keywords:** Non-small cell lung cancer, Symptomatic brain metastasis, EGFR mutation, Third-generation EGFR-TKIs, Upfront local therapy, Survival outcome

## Abstract

**Background:**

The third-generation epidermal growth factor receptor tyrosine kinase inhibitors (EGFR-TKIs) can penetrate blood–brain barrier and are effective for brain metastases (BMs). There is no consensus on the optimal sequence of local therapy (LT) and EGFR-TKIs for symptomatic BM patients because patients suffering neurological symptoms were not enrolled in most clinical trials.

**Methods:**

Non-small cell lung cancer (NSCLC) patients with EGFR mutation (EGFRm) and symptomatic BM receiving first-line osimertinib and aumolertinib from two medical centers were collected. All participants were allocated into the third-generation EGFR-TKIs (TKIs) group and the upfront LT (uLT) plus third-generation EGFR-TKIs (TKIs + uLT) group. Demographic data, survival outcomes, treatment failure patterns, and adverse events were evaluated between the two groups. We also conducted subgroup analyses to explore the impact of BM number on survival outcomes.

**Results:**

86 patients were enrolled, 44 in the TKIs group and 42 in the TKIs + uLT group. There were no significant differences in the short-term response between the groups. TKIs + uLT was associated with significantly longer overall survival (OS) (43 vs. 28 months; hazard ratio [HR], 0.36, 95% confidence interval [CI], 0.17–0.77; *p* = .011). No differences in progression-free survival (PFS), intracranial PFS (iPFS), failure patterns, or safety were observed. In subgroup analyses of oligo-BM patients, TKIs + uLT could prolong OS (43 vs. 31 months; HR 0.22; 95% CI 0.05–0.92; *p* = .015).

**Conclusions:**

EGFRm NSCLC patients with symptomatic BM might benefit from uLT, particularly oligo-BM patients. However, larger prospective cohort studies should be carried out to confirm the responses of the TKIs + uLT scheme.

**Supplementary Information:**

The online version contains supplementary material available at 10.1007/s00432-023-05538-9.

## Introduction

Lung cancer represents one of the most common tumors and the leading cause of cancer deaths, contributing to 21% of cancer deaths in 2022 in the United States (Siegel et al. 2022). Approximately 80–85% of lung cancer cases are non-small cell lung cancer (NSCLC) (D'Addario et al. 2010). Brain metastasis (BM), a frequent and classically devastating complication, occurs in nearly 25–40% of NSCLC patients (Barnholtz-Sloan et al. 2004). Previous extensive studies reported that the incidence of BM elevates among NSCLC patients harboring driver gene epidermal growth factor receptor (EGFR) mutation (Shin et al. 2014).

Local therapy (LT), such as whole-brain radiotherapy (WBRT), stereotactic radiosurgery (SRS), and surgical resection is the current mainstay of BM treatment, owing to the poor penetration of large molecule chemotherapeutic agents through the blood–brain barrier (BBB) (Wilhelm et al. 2013; Zhai et al. 2021). However, EGFR-tyrosine kinase inhibitors (EGFR-TKIs), with a higher BBB permeation rate, have been shifting the treatment paradigm (Park et al. 2012). Third-generation EGFR-TKIs have a higher permeation ratio and specific inhibition than first- or second-generation EGFR-TKIs, inhibiting both EGFR classic mutations (EGFR 19del and EGFR L8585R), and acquired T790M-induced resistance thus would be a promising treatment strategy for NSCLC with BM (Cross et al. 2014). In preclinical research, it has been suggested that osimertinib presented greater penetrating capacity of the mouse BBB than first- or second-generation EGFR-TKIs (Ballard et al. 2016). Osimertinib also showed remarkable efficacy with a higher intracranial response rate and a longer intracranial progression-free survival (iPFS) in some clinical trials. In a multi-center phase III clinical trial (FLAURA), osimertinib first-line treatment for advanced NSCLC with BM is associated with prolonging a median progression-free survival (PFS) up to 15.2 months and higher potency in comparison with gefitinib or erlotinib (HR = 0.47, 95% confidence interval [CI], 0.30–0.74) (Soria et al. 2018). Aumolertinib is a novel third-generation EGFR-TKI approved in China and is commonly used as first-line therapy for BM NSCLC (Lu et al. 2022). Compared with gefitinib, aumolertinib was reported to reduce the risk of disease progression and prolong PFS in BM patients (15.3 months and 8.2 months, HR = 0.38; *p* < 0.0001) (Lu et al. 2022). Unfortunately, clinical trials to date have tended to focus on untreated, or stable BM patients rather than those with untreated symptomatic BMs (Soria et al. 2018; Mok et al. 2017).

According to the European Association of Neuro-Oncology-European Society for Medical Oncology (EANO-ESMO) and the American Society for Clinical Oncology-Society for Neuro-Oncology-American Society for Radiation Oncology (ASCO-SNO-ASTRO) guidelines, EGFRm NSCLC patients with asymptomatic or oligo symptomatic BMs were recommended to use EGFR-TKIs without radiotherapy (Rhun et al. 2021; Vogelbaum et al. 2022). On the other hand, for patients with symptomatic BMs, uLT is recommended (radiosurgery or radiation therapy and/or surgery) as LT shows great potential for instant alleviation of neurological symptoms among them, indicating a novel BM management strategy of LT plus EGFR-TKIs (Rhun et al. 2021; Vogelbaum et al. 2022; Yang et al. 2017; Ni et al. 2019; Bhandari et al. 2021; Gondi et al. 2022). In a large survey from Chinese oncologists, the participants were unanimous in the view that radiotherapy (RT) plus TKIs were the preferred regimen for EGFRm NSCLC patients with BM when neurological symptoms presented (Yu et al. 2022). The synergistic effects between EGFR-TKIs and RT have been proved in previous studies (Zeng et al. 2015; Kong et al. 2017). The disruption of RT to BBB increases the TKIs permeability, and TKIs lower the radiation resistance of overexpressed EGFR wild-type cells while elevating EGFR-mutant cells’ sensitivity to RT (Kong et al. 2017; Khalifa et al. 2016). The clinical value of LT plus EGFR-TKIs for BM patients remains controversial. Although efficacy between RT and RT plus EGFR-TKIs has been compared by previous studies, a systematic understanding of how LT in combination with third-generation EGFR-TKIs acts on BM patients, especially symptomatic ones is still lacking (Zhai et al. 2021; Zhao et al. 2022; Xie et al. 2019; Yu et al. 2021). This study seeks to obtain data which will help explore the optimal sequence of LT for symptomatic BM patients in the era of first-line third-generation EGFR-TKIs.

Herein, we comprehensively performed a multi-institution retrospective study to compare survival outcomes between third-generation EGFR-TKIs (osimertinib and aumolertinib) with and without LT to identify the optimal sequence and explore the safety and effectiveness of third-generation EGFR-TKIs plus LT in real-world symptomatic BM patients with EGFR-mutant cohort.

## Method

### Study design and patients

The present study retrospectively collected EGFR-mutant NSCLC patients with symptomatic BM treated with first-line osimertinib or aumolertinib from 2018 to 2022 at Xiangya hospital and Xiangxi Autonomous Prefecture People’s Hospital. The specific inclusion criteria are as follows: (1) Age > 18 years old; (2) histologically or cytologically confirmed NSCLC without the second primary tumor and harboring common EGFR mutations (19del or L858R); (3) BM was confirmed by magnetic resonance imaging (MRI) at the initial diagnosis; (4) with neurological symptoms; (5) receiving first-line osimertinib or aumolertinib at least one month; (6) with complete clinical data and regular follow-up containing brain MRI; and (7) with measurable CNS lesions. Based on the inclusion criteria, a total of 87 patients were included in this study.

All patients were assigned to two groups according to the various interventions: third-generation EGFR-TKIs alone (TKIs) and third-generation EGFR-TKIs plus uLT (TKIs + uLT). All patients received first-line treatment with oral osimertinib (AZD9291, 80 mg × 30 tablets/package, AstraZeneca, UK), 80 mg orally, once a day, or aumolertinib (HS-10296, 55 mg × 20 tablets/package, Jiangsu Haosen Pharmaceutical Co. Ltd. China), 110 mg orally, once a day until disease progression or intolerable adverse reactions. The term “uLT” pertains to the administration of LT subsequent to diagnosis but prior to disease progression in patients undergoing first-line third-generation EGFR-TKIs treatment. LT modalities comprise SRS (Gamma knife radiosurgery), WBRT, and surgical resection. SRS was recommended for oligo-BMs, usually defined as 1–3 BM lesions (Vogelbaum et al. 2022; Gondi et al. 2022). WBRT was used in multiple BMs patients (Vogelbaum et al. 2022; Gondi et al. 2022). Surgical procedures were evaluated by neurosurgeons according to the status of BMs (Vogelbaum et al. 2022; Gondi et al. 2022). Altogether, treatment strategies were determined by the physician with full consideration of patient’s wishes. Most patients received 30 Gy of WBRT (10 fractions of 3 Gy). And SRS was performed following the Radiation Therapy Oncology Group (RTOG) guidelines. Patients treated with WBRT and SRS were assigned to the RT group.

The clinical characteristics include age, sex, dates of initial cancer diagnosis, smoking history, EGFR mutations types, number, location, and size of cranial metastasis, status of extracranial organs metastases, time of initial osimertinib or aumolertinib treatment, time and model of RT, imaging data of follow-up, adverse events (AEs), time and patterns of treatment failure, survival status, and death time. The current study was performed following the Helsinki Declaration. It was approved by the Institutional Review Board of Xiangya Hospital.

### Assessment of response and endpoints

An assessment of the response was conducted with the Response Evaluation Criteria in Solid Tumors (RECIST) 1.1 guideline, 4–6 months after treatment. Radiology scans including brain MRI and chest, abdomen, and pelvis computed tomography (CT) were used to evaluate intracranial and systemic responses. The first reexamination of brain MRI and chest CT was performed 1 month after osimertinib or aumolertinib treatment. During the disease course, brain MRI and chest CT were performed every 3 months to observe the changes in brain metastases and chest lesions. In the current study, overall survival (OS), PFS, and intracranial progression-free survival (iPFS) were regarded as primary endpoints. OS is defined as the time between osimertinib or aumolertinib initiation to death from any cause. PFS referred to the time from the initiation of osimertinib or aumolertinib treatment to the date of disease progression, death, or the last follow-up visit. iPFS was defined as the time from osimertinib or aumolertinib treatment until BM progression. Secondary endpoints were objective response rate (ORR), disease control rate (DCR), and safety. DCR included complete response (CR), partial response (PR), or stable disease (SD), and ORR was CR plus PR. Safety was evaluated according to the National Cancer Institute Common Terminology Criteria for Adverse Events (CTCAE) version 4.0. Common AEs mainly include diarrhea, rash, oral ulcer, paronychia, nausea, leukopenia, hepatobiliary disorder, and leukoencephalopathy. The clinical severity of white matter lesions was evaluated based on the Fazekas scale: absent lesions (grade 0), multiple punctate lesions (grade 1), beginning confluency of lesions (grade 2), and large confluent lesions (grade 3) (Fazekas, et al. 1987). The tumor volume was determined by delineating the tumor region on each MRI scan using Varian software, both at baseline and after 4–6 months of treatment. In the assessment of progressive disease (PD), the patterns included lung in situ progression (LPD), extracranial metastasis progression (EPD), intracranial progression (IPD), and multiple progressions mixed.

### Statistics analysis

The distributions of patients’ baseline characteristics and tumor size change in two groups were compared using *t* test or Mann–Whitney for continuous variables and the Chi-square test or Fisher’s exact test for categorical variables. The comparisons of tumor responses, treatment failure patterns, and AEs in the two groups were analyzed using the Chi-square test or Fisher’s exact test. The Kaplan–Meier method was performed to compare the survival curves (OS, PFS, iPFS) of two groups (TKIs and TKIs + uLT) and subgroups. The difference in survival outcome was analyzed using the log-rank test and Cox regression model in all patients. All analyses were done with R (version 4.2.1) and SPSS software (version 26.0). The two-tailed *p* value < 0.05 was considered statistically significant.

## Results

### Patients’ baseline characteristics

**Fig. 1:**
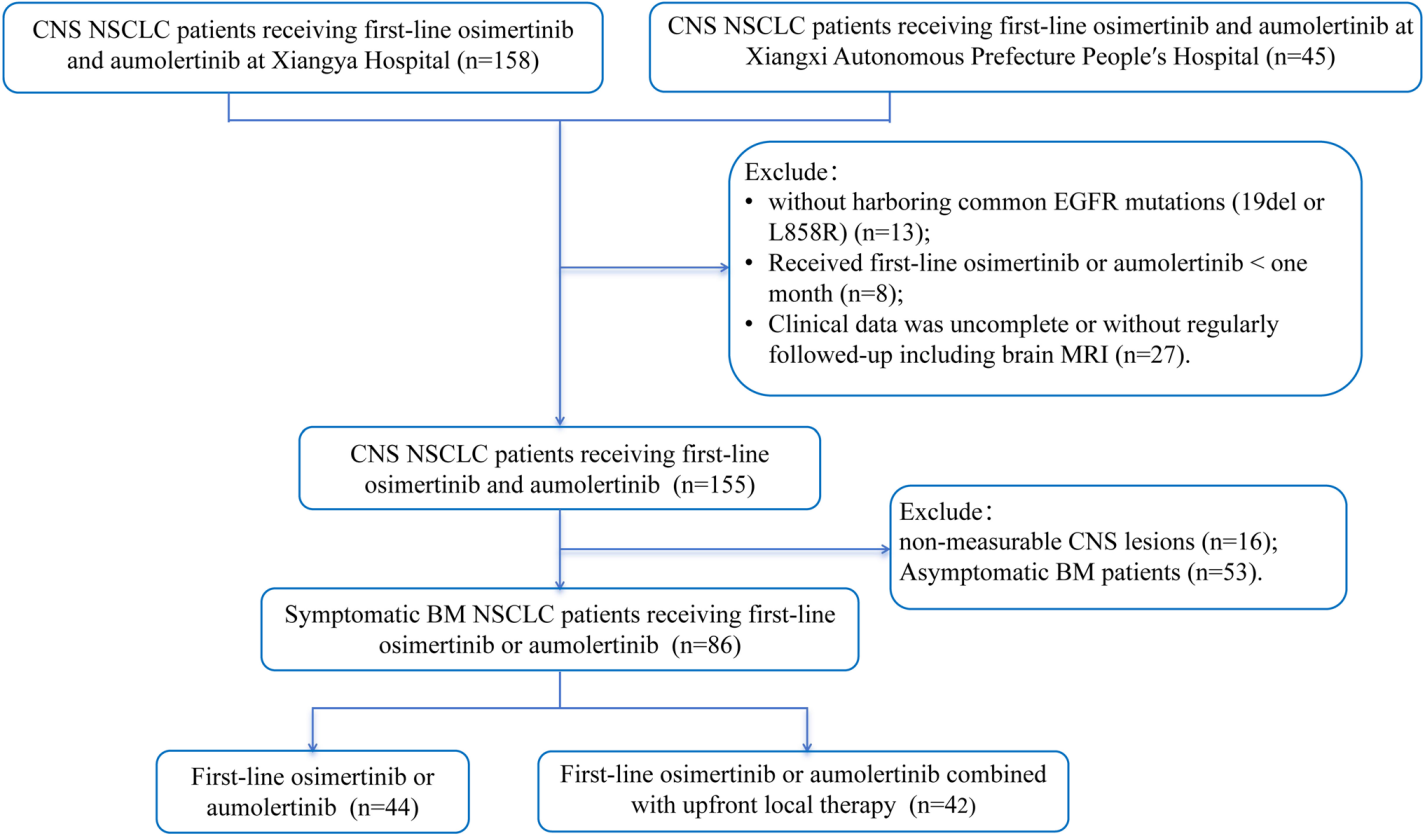
The participant flowchart

After the screening, 86 symptomatic BM patients who met the selection criteria were enrolled (Fig. 1). Among them, 44 (51.2%) patients were treated with first-line third-generation EGFR-TKIs alone (TKIs group) and 42 (48.8%) patients received first-line third-generation EGFR-TKIs plus uLT (TKIs + uLT group). Among upfront local regimens, 14 (33.33%) patients received surgery and 28 (66.67%) patients were treated with cranial RT (WBRT = 14, SRS = 14). Among the patients who were treated with radiotherapy, a total of 17 individuals received concurrent treatment of radiotherapy and EGFR-TKIs, while 11 patients underwent EGFR-TKIs treatment prior to radiotherapy. Concurrent radiotherapy denotes the administration of EGFR-TKIs at the beginning of cranial radiotherapy, while sequential radiotherapy involves the administration of EGFR-TKIs treatment prior to the initiation of radiotherapy (Zhai et al. 2021). The duration between the commencement of EGFR-TKIs and the initiation of radiotherapy varied, spanning from 1 week to 10 months, with the majority of instances concentrated within the 1–4-month period (*n* = 7). In the case of patients who underwent surgical intervention, all individuals underwent surgery as the initial treatment modality, followed by administration of EGFR-TKIs medication within a timeframe of 1 week to 2 months post-surgery. Additionally, 4 patients received localized irradiation specifically targeting the tumor bed subsequent to their surgical procedure.

The baseline characteristics of all patients are shown in Table 1 and Figure S1. All patients were administered first-line third-generation EGFR-TKIs treatment for an average duration of 16 months, with a range of 1–49 months. The duration of third-generation EGFR-TKIs treatment did not exhibit any statistically significant difference between the two groups (*p* = 0.362). No statistically significant difference was found between two groups in most characteristics, such as sex, age, mutation type, smoking status, extracranial metastasis, and BM max diameter. In the TKIs group, 15 (34.09%) patients were male, while 29 (65.91%) were female. In the TKIs + uLT group, the gender distribution is similar. Females had a relatively large proportion (59.52%) and 40.48% were male. In the two groups, there were 59.09% and 54.76% of subjects older than 55, respectively. As for the status of driver gene mutation, distributions of 19del and L858R in each of the two groups were similar. 34 (77.27%) patients in the TKIs group and 31 (73.81%) patients in the TKIs + uLT group were non-smokers. The rate of extracranial metastasis patients was 61.36% in the TKIs group and 47.62% in the TKIs + uLT group. Most of the BM in both groups were less than 30 mm in diameter (79.55% vs. 66.67%).

**Table 1:** Baseline characteristics of patients

	TKIs (*n* = 44)	TKIs + uLT (*n* = 42)	*p*
Sex (*n*, %)			**0.697**
Male	15 (34.09%)	17 (40.48%)	
Female	29 (65.91%)	25 (59.52%)	
Age (*n*, %)			**0.851**
≤ 55	18 (40.91%)	19 (45.23%)	
> 55	26 (59.09%)	23 (54.76%)	
Mutation (*n*, %)			**1.000**
L858R	18 (40.91%)	18 (42.86%)	
19del	26 (59.09%)	24 (57.14%)	
Smoking (*n*, %)			**0.902**
No	34 (77.27%)	31 (73.81%)	
Yes	10 (22.73%)	11 (26.19%)	
BM number (*n*, %)			**0.053**
Oligo	16 (36.36%)	25 (59.52%)	
Extensive	28 (63.64%)	17 (40.48%)	
Extracranial metastasis (*n*, %)			**0.288**
No	17 (38.64%)	22 (52.38%)	
Yes	27 (61.36%)	20 (47.62%)	
BM max diameter (n, %)			**0.269**
≤ 30 mm	35 (79.55%)	28 (66.67%)	
> 30 mm	9 (20.45%)	14 (33.33%)	
Upfront local therapy (*n*, %)			–
Surgery	–	14 (33.33%)	
WBRT	–	14 (33.33%)	
SRS	–	14 (33.33%)	
Duration of TKIs [median (Q1, Q3)]	14.5 (7.75, 19)	14.5 (11.25, 21.75)	**0.362**

*TKIs* tyrosine kinase inhibitors, *TKIs* + *uLT* tyrosine kinase inhibitors plus upfront local therapy, *BM* brain metastasis, *WBRT* whole-brain radiotherapy, *SRS* stereotactic radiosurgery, *Oligo-BM* 1–3 BM lesions, *Extensive BM* > 3 BM lesions

In this study, all patients presented symptomatic BM. The most common neurological symptoms included headache (61.63%), dizziness (45.35%), altered mental status (17.44%), limb numbness or weakness (15.12%), vomiting (12.79%), epilepsy (4.65%), and walking instability (4.65%). Furthermore, 2 (2.33%) patients experienced hemiplegia and 1 (1.16%) patient experienced choking. The detailed information is presented in Table S1. The majority of symptomatic patients (*n* = 59, 68.6%) who were primarily associated with perilesional edema or a mass space-occupying effect, as evidenced by the MRI. Additionally, a small number of patients (*n* = 4, 4.7%) demonstrated high intracranial pressure without apparent imaging manifestations, as revealed by lumbar puncture. The majority of hospitalized patients (*n* = 60, 69.8%) received treatment involving the administration of mannitol, glycerin fructose, corticosteroids, or other medications aimed at alleviating symptoms (Table S2).

### Response evaluation

In this study, follow-up is complete through March 1, 2023 and the median duration follow-up time was 17.0 months (range 1.0–53.0 months). At the time of data cut-off, 27 (31%) patients died, and 59 (69%) patients were still alive. All patients were evaluated for the therapeutic effect after 4–6 months of third-generation EGFR-TKIs treatment. The changes in systemic and brain tumor diameter are shown in Figure S2. ORR was 62.79%, DCR was 98.84%, intracranial ORR (iORR) was 60.47%, and intracranial DCR (iDCR) was 90.70% in the whole population (Table 2). In the TKIs group, none of the patients obtained a CR or PD. ORR was 59.09%, and DCR was 100%, including 26 patients with PR and 18 patients with SD. iORR was 59.09%, including 4 patients with intracranial CR. And iDCR could reach 88.64%. In the groups receiving TKIs + uLT, 1 patient reached CR and 8 patients reached intracranial CR. The rates of ORR and iORR were found to be higher compared to the group receiving only TKIs. However, these differences did not reach statistical significance (ORR, 66.67% vs 59.09%, *p* = 0.615; iORR, 61.90% vs 59.09%, *p* = 0.763). Similarly, there was no significant difference observed in DCR and iDCR between the two groups (DCR, 97.62% vs 100%, *p* = 0.981; iDCR, 92.86% vs 88.64%, *p* = 0.763).

**Table 2:** Response in two groups

	ITT (*n* = 86)	TKIs (*n* = 44)	TKIs + uLT (*n* = 42)	*p*
CR, *n* (%)	1 (1.16%)	0 (0%)	1 (2.38%)	0.981
PR, *n* (%)	53 (61.63%)	26 (59.09%)	27 (64.29%)	0.785
SD, *n* (%)	31 (36.05%)	18 (40.91%)	13 (30.95%)	0.461
PD, *n* (%)	1 (1.16%)	0 (0%)	1 (2.38%)	0.981
ORR	62.79%	59.09%	66.67%	0.615
DCR	98.84%	100%	97.62%	0.981
iCR, *n* (%)	12 (13.95%)	4 (9.09%)	8 (19.05%)	0.307
iPR, *n* (%)	40 (46.51%)	22 (50.00%)	18 (42.86%)	0.654
iSD, *n* (%)	26 (30.23%)	13 (29.55%)	13 (30.95%)	1.000
iPD, *n* (%)	8 (9.30%)	5 (13.36%)	3 (7.14%)	0.763
iORR	60.47%	59.09%	61.90%	0.963
iDCR	90.70%	88.64%	92.86%	0.763

*ITT* intention-to-treat population, *CR* complete response, *PR* partial response, *SD* stable disease, *PD* progressive disease, *ORR* objective response rate, *DCR* disease control rate, *iCR* intracranial complete response, *iPR* intracranial partial response, *iSD* intracranial stable disease, *iPD* intracranial progressive disease, *iORR* intracranial objective response rate, *iDCR* intracranial disease control rate

## Survival outcome

### Survival outcomes in the whole population

**Fig. 2:**
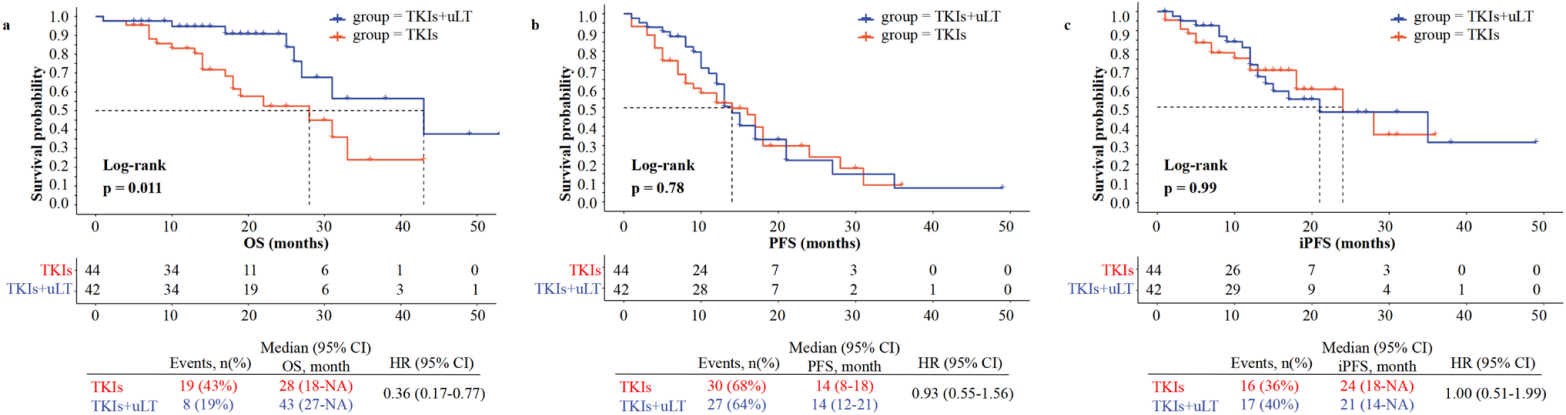
OS **a**, PFS **b**, iPFS **c** of all patients. *HR* hazard ratio, *CI* confidence interval, *NA* not available

With uLT, there was a highly significant prolongation of OS compared with TKIs alone. The median OS was 43 months (95% CI 27-NA) and 28 months (95% CI 18-NA) in the TKIs group and TKIs + uLT group, separately (hazard ratio [HR] 0.36; 95% CI 0.17–0.77; *p* = 0.011) (Fig. 2a). In the TKIs group, the median PFS was 14 months (95% CI 8–18). And in the TKIs + uLT group, the median PFS was 14 months (95% CI 12–21), and there were no significant differences in PFS between the two groups (HR 0.93; 95% CI 0.55–1.56; *p* = 0.78) (Fig. 2b). Additionally, iPFS was also similar for patients in the two groups (HR 1.00; 95% CI 0.51–1.99; *p* = 0.99). Median iPFS in TKIs group was 24 months (95% CI 18-NA) and in TKIs + uLT groups was 21 months (95% CI 14-NA) (Fig. 2c).

### Subgroup analyses

**Fig. 3:**
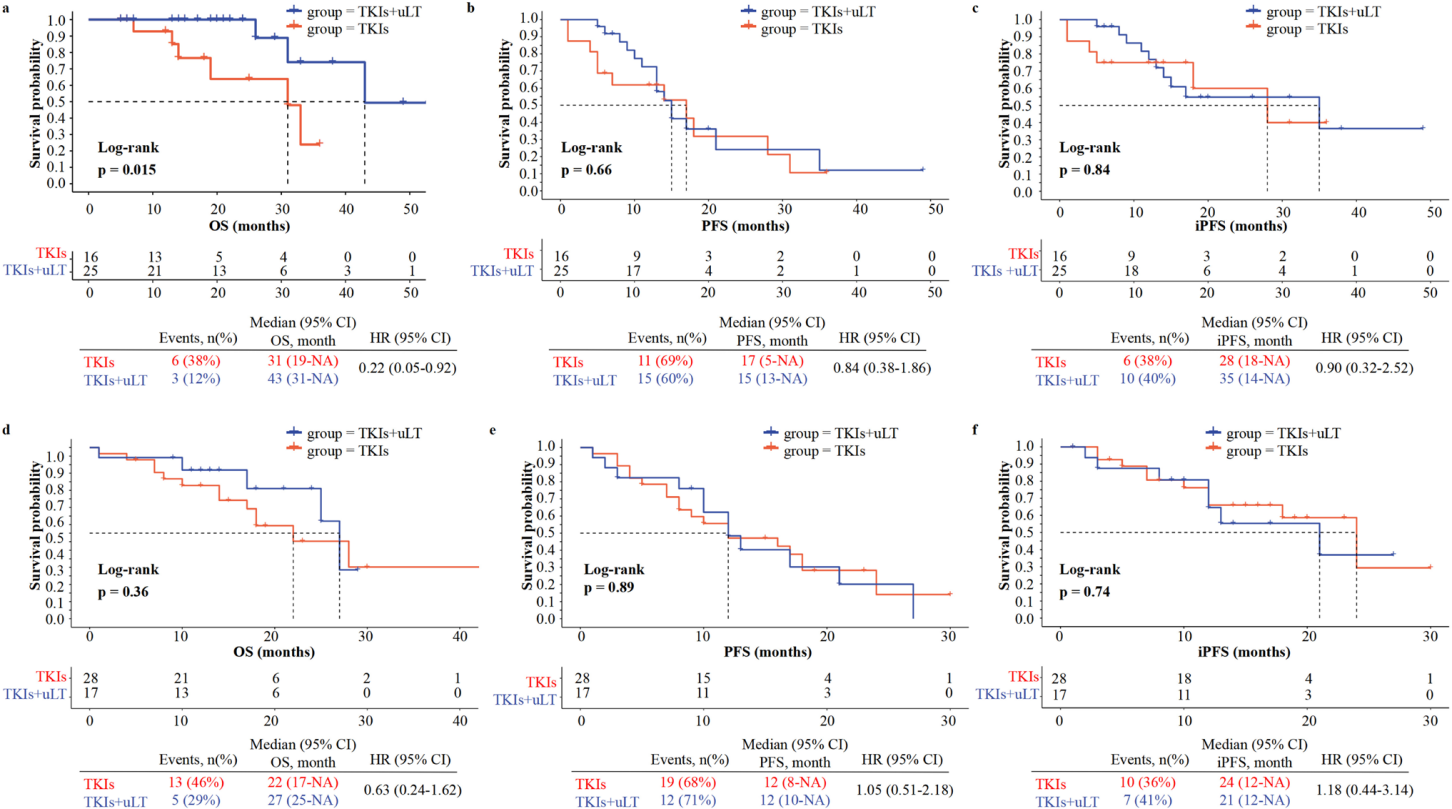
OS, PFS, iPFS of patients stratified via BM lesions’ number. **a**–**c** OS, PFS, and iPFS of oligo-BM patients; **d**–**f** OS, PFS, and iPFS of extensive BM patients. *BM* brain metastasis, *HR* hazard ratio, *CI* confidence interval, *NA* not available

Subgroup analyses were then conducted for OS, PFS, and iPFS, and the detailed result was elucidated in Fig. 3. All patients were divided into the oligo subgroup (Fig. 3a–c) and extensive BM groups (Fig. 3d–f) according to the number of BM lesions. In the oligo-BM cohort (Fig. 3b, c), the inclusion of uLT did not yield a significant effect on PFS or iPFS (HR 0.90; 95% CI 0.32–2.52; *p* = 0.84). The result of the OS was similar to this for all populations, and the OS of patients receiving TKIs + uLT was notably longer than that of the other group (Fig. 3a; 43 vs. 31 months; HR 0.22; 95% CI 0.05–0.92; *p* = 0.015). Conversely, in the extensive BM cohort, uLT exhibited minimal influence on survival outcomes. Moreover, there was no statistical significance observed in the OS, PFS, or iPFS between the two groups; however, a trend toward improved OS was evident in the TKIs + uLT group (HR 0.63, 95% CI 0.24–1.62; *p* = 0.36) (Fig. 3e, f).

### The pattern of treatment failure

**Fig. 4:**
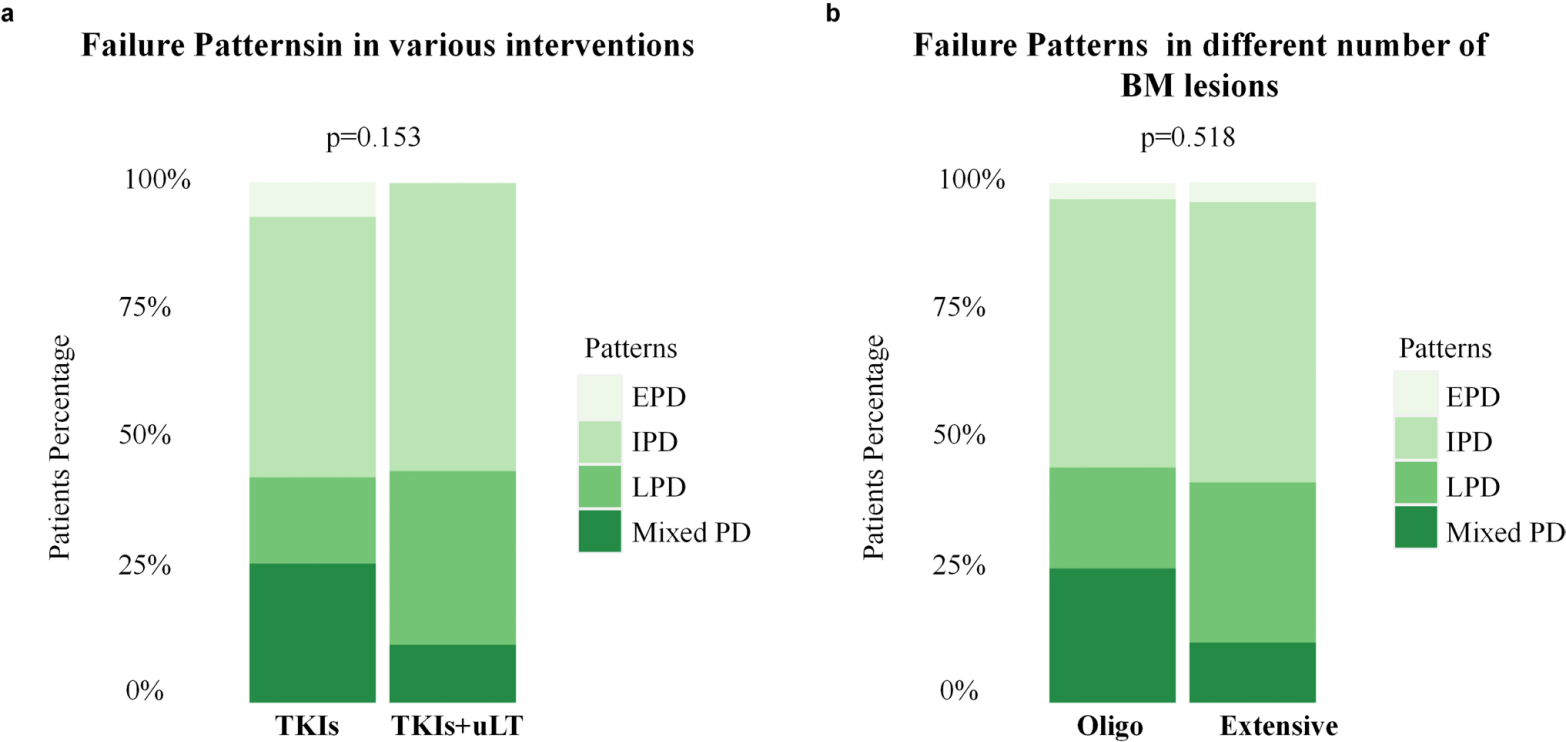
Treatment failure patterns. *BM* brain metastasis, *EPD* extracranial metastasis progression, *IPD* intracranial progression, *LPD* lung in situ progression, *Mixed PD* multiple progressions mixed

At the time of data cut-off, 57 (66%) patients had experienced PD. The time-receiving regimens and response status of each patient are displayed in Figure S3. The main sites of PD were lung (25%), brain (53%), bone (2%), liver (2%), and mixed (19%). In the TKIs group, a total of 30 patients had PD. LPD, IPD, EPD, and mixed PD occurred in 5 (17%), 15 (50%), 2 (7%), and 8 (27%) patients, respectively. In the TKIs + uLT group, 27 patients suffered PD including 15 (56%) IPD, 9 (33%) LPD, and 3 (11%) mixed PD. Among them, IPD accounts for a large proportion of the two groups (50% and 56%). Furthermore, compared to the TKIs group, a greater proportion of patients had only lung progression in the TKIs + uLT group (33% vs. 17%). See Table S4 for details. The percentage of patients developing IPD, LPD, EPD, and mixed PD was not significantly different in the results of patients receiving third-generation EGFR-TKIs alone and those receiving third-generation EGFR-TKIs combined with uLT (*p* = 0.153, Fig. 4a). Table S5 and Fig. 4b also demonstrate that there were no apparent differences in the patterns of treatment failure between the oligo and extensive BM groups and the main failure pattern was IPD (54% and 52%).

### Changes in tumor volume

A total of 50 patients’ brain MRI scans, including both baseline and post-treatment scans conducted after a period of 4–6 months, were obtained and subjected to manual delineation in order to determine the overall tumor volume. However, due to lack of MRI images, data extraction was not feasible for 36 patients. Among 50 patients, the baseline tumor volumes exhibited a range of 0.03 to 93.4 cm^3^, with a mean of 18.79 cm^3^. Following a treatment period of 4–6 months, the tumor volumes ranged from 0 to 41.7 cm^3^, with a mean of 3.77 cm^3^. The alterations in tumor volume displayed a range of -86 to 100%, with a mean change of 72.65%. Detailed information regarding the characteristics of tumor volume changes in various subgroups is shown in Tables S6 and S7. There was no statistically significant difference observed in either the various interventions or the different number of BM lesions groups (*p* = 0.921, *p* = 0.152).

### Safety

The AEs are summarized in Table 3. AEs were reported in 29 patients (66%) in the TKIs group and 28 (67%) in the TKIs + uLT group. There was no significant difference in adverse reactions between the two groups (*p* = 1.000). In all patients, the most common AEs were rash (30% in the TKIs group and 31% in the TKIs + uLT group), leukopenia (36% and 19%, respectively), and diarrhea (9% and 14%, respectively). Furthermore, oral ulcers occurred in 5% of patients with TKIs and 14% of patients with TKIs + uLT. Paronychia occurred in 11% of patients with TKIs and 2% of patients with TKIs + uLT. Hepatobiliary disorder occurred in 14% of patients with TKIs and 12% of patients with TKIs + uLT. Nausea occurred in 9% of patients with TKIs and 7% of patients with TKIs + uLT. Leukoencephalopathy occurred in 5% of patients with TKIs and 17% of patients with TKIs + uLT (Table 3). Grade ≥ 3 AEs were reported in 4 patients (9%) in the TKIs group and in 5 (12%) in the TKIs + uLT group. The most common grade ≥ 3 AEs were rash (2% vs. 2%), diarrhea (2% vs. 2%), leukopenia (0% vs. 5%), and hepatobiliary disorder (0% vs. 2%), respectively (Table 3).

**Table 3:** Summary of adverse effects

AEs, *n* (%)	TKIs (*n* = 44)	TKIs + uLT (*n* = 42)	*p*
Any grade	29 (65.91%)	28 (66.67%)	1.000
Rash	13 (29.55%)	13 (30.95%)	1.000
Leukopenia	16 (36.36%)	8 (19.05%)	0.121
Diarrhea	4 (9.09%)	6 (14.29%)	0.678
Oral ulcer	2 (4.55%)	6 (14.29%)	0.237
Paronychia	5 (11.36%)	1 (2.38%)	0.226
Hepatobiliary disorder	6 (13.64%)	5 (11.90%)	1.000
Nausea	4 (9.09%)	3 (7.14%)	1.000
Leukoencephalopathy	2 (4.55%)	7 (16.65%)	0.138
Grade ≥ 3	4 (9.09%)	5 (11.9%)	0.942
Leukopenia	2 (4.55%)	0 (0%)	0.495
Hepatobiliary disorder	0	1 (2.38%)	0.981
Leukoencephalopathy	0	2 (4.76%)	0.454
Rash	1 (2.27%)	1 (2.38%)	1.000
Diarrhea	1 (2.27%)	1 (2.38%)	1.000

## Discussion

Currently, there is no consensus on treatment for EGFR mutation-positive patients with symptomatic brain metastases. In accordance with the guidelines set forth by the National Comprehensive Cancer Network (NCCN), it is recommended that patients receiving third-generation EGFR-TKIs who experience CNS progression should be considered for the continuation of third-generation EGFR-TKIs and/or local LT (National Comprehensive Cancer Network 2023). However, LT (radiosurgery or radiation therapy and/or surgery) was recommended regardless of the systemic therapy used for the systemic disease by the guideline of EANO-ESMO and ASCO-SNO-ASTRO for symptomatic brain metastases (Rhun et al. 2021; Vogelbaum et al. 2022). Although some previous studies had explored the efficacy of the TKIs with or without uLT in EGFRm patients with BM, there were some weaknesses and limitations as follows (Zhai et al. 2021; Zhao et al. 2022; Yu et al. 2021; Langston et al. 2023) (Table 4): (i) The study population has no detailed distinction with first-, second-, and third-generation TKIs being used at the same time. Large heterogeneity existed among included patients. (ii) There were limited data or analysis on symptomatic BM patients. (iii) The subgroup analysis of different numbers of BM lesions was unable to carry out due to small sample size. To the best of our knowledge, the current study is the first work studying survival outcomes of advanced EGFRm patients with symptomatic BM receiving first-line third-generation EGFR-TKIs with or without uLT. It also focuses on treatment failure patterns and safety and describes the whole course of the disease.

**Table 4:** Summary of different studies exploring third-generation EGFR-TKIs therapeutic strategy in EGFRm patients with BM

References	Patients	Control	Intervention	Center	Subgroup	Outcome
Yu et al. (2021)	205	First- and second-line osimertinib	First- and second-line osimertinib plus upfront RT	Two	Oligo-BM vs. multiple-BM	OS, PFS, iPFS, iTTP
Zhao et al. (2022)	367	First-line EGFR-TKIs (gefitinib, erlotinib and osimertinib)	First-line EGFR-TKIs plus uLT	One	First-generation EGFR-TKIs vs. osimertinib	OS, PFS, iPFS, ORR
Zhai et al. (2021)	61	First-, second-, third-line osimertinib	Osimertinib plus RT	One	EGFR 19del vs. L858R	OS, PFS, iPFS, ORR, DCR
Langston et al. (2023)	12	First-, second-, third-line EGFR-TKIs (brigatinib, entrectinib, lorlatinib, alectinib, and osimertinib)	None	One	None	OS, CNS response, BM volume

*iTTP* the time to intracranial progression

The results of the present study revealed that headache, dizziness, limb numbness or weakness, altered mental status, vomiting, and epilepsy are the most frequent neurological symptoms. The ORR, iORR, and DCR of the combination therapy group were higher than those of the EGFR-TKIs monotherapy group, but the difference was not statistically significant. Further evaluations were done for long-term efficacy. Significantly higher OS was observed (43 months vs. 28 months; HR 0.36; 95% CI 0.17–0.77; *p* = 0.011) in the third-generation EGFR-TKIs plus uLT cohort when compared to third-generation EGFR-TKIs monotherapy patients. Among oligo-BM patients, TKIs + uLT also could improve median OS (43 vs. 31 months; HR 0.22; 95% CI 0.05–0.92; *p* = 0.015). The trends of the OS curves in the multi-BM subgroup showed that the addition of uLT was relatively better. Our data demonstrated that compared to TKIs monotherapy, the TKIs plus uLT statistically extended OS but not iPFS or PFS.

As previously mentioned, previous studies on this topic have primarily been retrospective in nature, resulting in varying and inconclusive findings. Zhao et al. discovered that in patients receiving first-generation TKIs, the use of uLT was associated with a longer OS. However, in patients treated with first-line osimertinib, there was no statistically significant difference in OS between those who received uLT and those who did not (Zhao et al. 2022). Yu et al. also observed that the OS, PFS, and iPFS did not differ between the osimertinib alone group and the osimertinib plus upfront radiotherapy group. However, for patients with limited BM, the OS, PFS, and iPFS were significantly prolonged (Yu et al. 2021). Zhai et al. conducted the similar study that found no significant differences between the use of osimertinib alone and the combination of osimertinib with upfront radiotherapy. Additionally, in the subgroup of patients with the L858R mutation, the OS was significantly longer in the osimertinib plus upfront radiotherapy group compared to the osimertinib alone group (Zhai et al. 2021). A meta-analysis of 24 studies that examined the effectiveness of combining upfront brain radiation therapy (RT) with first-/second-generation EGFR-TKIs compared to using only first-/second-generation EGFR-TKIs (Song et al. 2023). The meta-analysis revealed that there was no significant discrepancy in the benefit of iPFS between the two groups, encompassing both asymptomatic and symptomatic patients. Furthermore, the OS of asymptomatic patients with brain metastases notably improved with the administration of upfront brain RT in conjunction with EGFR-TKIs. In our study, the discordance between early endpoints and OS may arise due to several factors as follows. (ii) The limited duration of follow-up and inadequate sample size can introduce statistical bias. (ii) As this study was a retrospective non-randomized trial, the accurate interpretation of PFS or OS is hindered by the inclusion of an external control, which introduces bias. (iii) There are differences in the baseline characteristics of patients, with a majority of those in the TKIs group exhibiting extensive BM and a majority of those in the uLT + TKIs group having limited BM. Subgroup analysis also indicates that uLT + TKIs may have a more favorable impact on patients with limited BM. This may have led to the uLT + TKIs group showing better outcomes in analysis. (iv) The subsequent therapeutic interventions included chemotherapy, immunotherapy, or radiotherapy, and no statistically significant distinction was observed between the two cohorts (Table S3). However, it is imperative to recognize the inherent limitations of the study's sample size, consisting of only 27 patients who received subsequent therapy after disease progression, which may not provide a precise representation of the influence of subsequent therapy on OS.

In addition, treatment failure patterns among both groups showed no visible difference. Both groups presented IPD predominance, and the addition of uLT could not modify the progression of the disease. While our study provided feasibility data and preliminary results, more comprehensive research and larger studies are required to further validate these results.

Preclinical studies suggested that third-generation EGFR-TKIs, such as osimertinib and aumolertinib could penetrate the BBB with higher drug concentration in the brain (Ballard et al. 2016; Talele 2016; Zhang et al. 2023). Although there is substantial evidence indicating new-generation EGFR-TKIs could significantly prolong PFS of patients with CNS metastasis compared to first-generation EGFR-TKIs, patients with baseline BM always showed poorer PFS than non-BM patients (FLAURA: 15.2 vs. 19.1 months; OS, AENEAS: 15.3 vs. 19.3 months) (Soria et al. 2018; Lu et al. 2022; Ramalingam et al. 2020). Additionally, in patients with baseline BM, after osimertinib treatment, CNS progression (*n* = 19) accounted for 34% of all progression patterns (*n* = 29) (Lu et al. 2022). Therefore, intracranial metastasis was regarded as a poor prognostic factor for the treatment of the third-generation EGFR-TKIs and the CNS is still the main site of PD. Zhu et al. also reported that compared to the first-generation EGFR-TKIs, osimertinib showed a similar proportion of patients who developed symptomatic CNS metastasis (8.0% vs. 10.4%) and could not change the patterns of PD (Zhao et al. 2022; Zhou, et al. 2020). Therefore, control of intracranial lesions may be an effective way to improve the survival of patients with baseline BM treated with third-generation EGFR-TKIs.

Currently, SRS or surgical resection for oligo-BM and WBRT for multiple BM are primary treatment options except for EGFR-TKIs. The therapeutic synergism between EGFR-TKIs and RT was observed in some studies in the past (Kong et al. 2017; Zhang et al. 2023). Researchers put forward that RT could destroy the BBB and increase its permeability (Nordal and Wong 2005). The EGFR signaling pathway also can be activated by RT to promote cell growth and DNA repair (Kang, et al. 2020; Ali, et al. 2022; Li et al. 2018). Similarly, EGFR-TKIs had a radiosensitizing effect by decreasing cell proliferation and suppressing the repair of DNA damage (Li et al. 2018; Welsh et al. 2013). Previous clinical trials suggested that compared with TKI monotherapy, brain RT may improve CNS ORR (64% vs. 34%) (Wu et al. 2018). In the era of the third-generation EGFR-TKIs; however, it is unclear that the optimal treatment combination or sequence of LT with EGFR-TKIs and patients of what characteristics benefit the most. Our results revealed that uLT may effectively prolong OS (*p* = 0.011), especially in oligo-BM patients (*p* = 0.015). These findings were similar to previous studies. Yu F, et al. found that SRS combined with osimertinib could improve the OS of patients with oligo-BM (*p* = 0.026) (Yu et al. 2021). Zhao Y, et al. and Miyawaki E, et al. observed similar results in patients treated with first-generation EGFR-TKIs (Zhao et al. 2022; Miyawaki et al. 2019). Zhao Y, et al. found upfront cranial LT is associated with prolonged OS, especially in SRS/Surgery (HR 0.58; 95% CI 0.37–0.91; *p* = 0.019) (Zhao et al. 2022). According to the results of Miyawaki E et al., in patients with 1–4 BMs, compared with the TKI group, the LT group showed significantly better OS (35 vs. 23 months; HR 0.54; 95% CI 0.32–0.90) (Miyawaki et al. 2019). In summary, for patients with a limited number of BM, uLT (SRS/Surgery) might improve their survival outcomes of them. In the future, large-scale and prospective study was warranted to validate our findings.

However, it remains questionable whether the combined strategy of targeted therapy with WBRT is superior to targeted therapy alone in patients with multiple BM, owing to the emergence of the third-generation EGFR-TKIs with higher intracranial activity. For multiple BMs, WBRT is still a traditional and standard first-line treatment, yet adverse events of WBRT, such as worse cognitive outcomes were common (Bhandari et al. 2021). A higher incidence of leukoencephalopathy in patients receiving WBRT or WBRT with a simultaneous integrated boost (SIB) (31.5%) than osimertinib alone (0%) was observed by Zhai and his colleagues (Zhai et al. 2021). With high rate of radiotherapy-related adverse events, searching for new treatment is necessary. However, the safety and effectiveness of using TKIs alone as a treatment method remain incompletely understood. A report including 9 patients with symptomatic BM showed most patients (67%) achieved intracranial objective responses after upfront use of the next-generation TKIs and proved upfront CNS-active TKIs may benefit patients with multifocal, large-volume, and symptomatic BM (Gal et al. 2020). Similar results supported that WBRT should be deferred or even withheld in another recent case report. Authors suggested that a multiple asymptomatic BM patient with EGFRm NSCLC treated with first-line osimertinib without RT reached iCR with the response of lung lesions (Ameku and Higa 2020). A case published in 2023 also reported an EGFRm NSCLC patient with symptomatic BM getting iCR after aumolertinib monotherapy (Shan et al. 2023). A retrospective study of 12 BM patients with ALK, EGFR, and ROS1-driven NSCLC described that extensive BM (> 10 BMs or leptomeningeal disease [LMD]) patients might achieve disease downstaging after receiving high CNS-active TKIs. They found that with newer-generation TKIs and close MRI follow-up, patients with extensive BM could reduce BM burden (number and size of brain tumor) and avoid WBRT. And at the time of CNS progression, salvage SRS can be considered (Langston et al. 2023). The findings of our study also indicate that tumor volume exhibited a significant reduction regardless of the presence or absence of uLT (*p* = 0.921). Moreover, patients with multiple BM lesions (more than three) did not experience any survival advantages from uLT. Although the published studies had a relatively small sample size, they offered a new treatment plan of upfront newer-generation TKIs combined with salvage SRS for multiple BM patients to avoid or delay WBRT and its side effects.

It has been recognized that radiotherapy, especially WBRT, can cause white matter lesions (Andrews et al. 2004; Li et al. 2007). A published study has reported the incidence of leukoencephalopathy after WBRT was 34.4% (Ebi et al. 2013). Whether third-generation TKI combined with radiotherapy will increase the incidence of white matter lesions still needs discussion. In our study, of the 9 patients with leukoencephalopathy, 8 received WBRT plus TKI. In the TKIs plus uLT group, grade 3 leukoencephalopathy was observed in 2 patients who were followed up for 24 months. Among 2 grade 3 leukoencephalopathy patients, the metastatic tumor of 1 patient treated with a gamma knife was located near the lateral ventricle. In the TKIs group, 1 patient had grade 3 leukoencephalopathy before treatment and did not aggravate after treatment. According to our results, TKIs alone do not appear to increase the risk of leukoencephalopathy.

There are several limitations in our study. First, owing to all data being collected retrospectively, it is difficult for us to get the precise time of neurological symptom alleviation. Such information is useful for evaluating the efficacy of treatment regimens. Second, our study lacks testing quality of life (QoL) and cognitive function between the two groups. Because BM also reduced QoL and cranial RT often causes cognitive function damage, evaluative observation of these values was important to treat BM and lower the burden of disease. Third, the subgroup analyses could not be explained adequately due to the small number of patients in the subgroup. Fourth, the duration of follow-up was short. Longer follow-up likely contributed to more credible results being detected.

Our study aims to explore and compare survival outcomes and the optimal sequence between third-generation EGFR-TKIs with or without uLT in real-world symptomatic BM patients with the EGFR-mutant cohort. We proposed a novel treatment strategy according to different BM lesions number. For oligo-BM patients, TKIs + uLT may benefit their survival outcomes without increasing side effects. As for multiple BM patients, upfront TKIs were used to reduce tumor burden (number and size), and RT target volume to lower radiation toxicity. For those insensitive lesions or oligo-progressive lesions, RT intervention was given. A treatment option involving upfront next-generation TKIs followed by salvaging SRS after progression could avoid WBRT to reduce late effects on neurocognitive function. Due to the small sample size and retrospective nature, the quality of evidence for these results remains limited. In the future, large-sample randomized controlled trials are warranted to validate our findings.

## Conclusion

Our study showed that NSCLC patients with EGFR mutation and symptomatic BM might benefit from uLT, particularly oligo-BM patients. Multiple BM patients can consider a treatment option involving upfront the third-generation EGFR-TKIs followed by RT. However, larger prospective cohort studies are required to validate these results.

## Clinical practice points

Currently, there exists a lack of agreement regarding the management of EGFRm patients presenting with symptomatic brain metastases. The objective of this study is to identify an optimal treatment approach for such patients. Our findings indicate that individuals with NSCLC and EGFR mutations, along with symptomatic brain metastases, may derive benefits from upfront local therapy, particularly those with limited brain metastases. For patients with multiple brain metastases, a treatment strategy involving initial administration of third-generation EGFR-TKIs followed by radiotherapy may be considered. These findings have the potential to offer valuable guidance to healthcare practitioners in the management of symptomatic brain metastases. Nevertheless, it is imperative that larger prospective cohort studies be conducted to validate these results.

## Supplementary Information

Below is the link to the electronic supplementary material.

Supplementary file1 (DOCX 2540 KB)

## Data Availability

The datasets used and analyzed during the current study are available from the corresponding author on reasonable request.

## Declarations

### Registry and the registration no. of the study/trial

N/A.

### Animal studies

N/A.
