# Supplementary material for: Optimal sequence of LT for symptomatic BM in EGFR-mutant NSCLC: a comparative study of first-line EGFR-TKIs with/without upfront LT
Source: J Cancer Res Clin Oncol. 2024 Feb 19;150(2):94. doi: 10.1007/s00432-023-05538-9 (PMC10874906; doi:10.1007/s00432-023-05538-9)
Supplement: Supplementary file 1 — Supplementary file1 (DOCX 2540 KB) [file 432_2023_5538_MOESM1_ESM.docx]

**Supplementary Online Content**

**Table S1.** Neurological symptoms in the whole population

**Table S2.** Some treatments to relieve symptoms

**Table S3.** Treatment options after progression

**Table S4.** Treatment failure patterns in various interventions

**Table S5.** Treatment failure patterns in different number of BM lesions

**Table S6.** Tumor volume assessment in various interventions

**Table S7.** Tumor volume assessment in different number of BM lesions

**Figure S1.** Characteristics of patients in two Groups

**Figure S2.** Waterfall plot for systemic tumor size (a) and CNS tumor size (b)

**Figure S3.** Treatment failure patterns in different number of BM lesions

**Table S1.** Neurological symptoms in the whole population

|  | ITT (n, %) | TKIs (n, %) | TKIs+uLT (n, %) |
| --- | --- | --- | --- |
| Headache | 53 (61.63%) | 28 (63.64%) | 25 (59.52%) |
| Dizziness | 39 (45.35%) | 20 (45.45%) | 19 (45.24%) |
| Altered mental status | 15 (17.44%) | 6 (13.64%) | 9 (21.43%) |
| Limb numbness or weakness | 13 (15.12%) | 9 (20.45%) | 4 (9.52%) |
| Vomiting | 11 (12.79%) | 3 (6.82%) | 8 (19.05%) |
| Epilepsy | 4 (4.65%) | 3 (6.82%) | 1 (2.38%) |
| Walking instability | 4 (4.65%) | 2 (4.54%) | 2 (4.76%) |
| Hemiplegia | 2 (2.33%) | 0 (0%) | 2 (4.76%) |
| Choking | 1 (1.16%) | 1 (2.27%) | 0 (0%) |

Abbreviation: ITT, intention-to-treat population.

**Table S2.** Some treatments to relieve symptoms

|  | ITT (n, %) | TKIs (n, %) | TKIs+uLT (n, %) |
| --- | --- | --- | --- |
| Mannitol or glycerin fructose | 47 (54.65%) | 23 (52.27%) | 24 (57.15%) |
| Corticosteroids | 42 (48.84%) | 13 (29.55%) | 29 (69.05%) |
| Antiepileptic drugs | 13 (15.12%) | 3 (6.82%) | 10 (23.81%) |
| Antipsychotic drugs | 1 (1.16%) | 1 (2.27%) | 0 (0%) |
| Ventriculoperitoneal shunting | 1 (1.16%) | 1 (2.27%) | 0 (0%) |
| Unclear | 26 (30.23%) | 17 (38.64%) | 9 (21.43%) |

Abbreviation: ITT, intention-to-treat population.

**Table S3.** Treatment options after progression

|  | ITT (n = 57) | TKIs (n = 30) | TKIs+uLT (n = 27) | p |
| --- | --- | --- | --- | --- |
| Chemotherapy | 9 (15.79%) | 6 (20.00%) | 3 (11.11%) | 0.579 |
| PD-1/PD-L1 | 1 (1.75%) | 1 (3.33%) | 0 (0%) | 1.000 |
| Chemotherapy + PD-1/PD-L1 | 7 (12.28%) | 3 (10.00%) | 4 (14.81%) | 0.882 |
| Anti-VEGF | 2 (3.51%) | 1 (3.33%) | 1 (3.70%) | 1.000 |
| Radiotherapy | 14 (24.56%) | 8 (26.67%) | 6 (22.22%) | 0.935 |
| Surgery | 1 (1.75%) | 0 (0%) | 1 (3.70%) | 0.958 |
| EGFR-TKI | 1 (1.75%) | 0 (0%) | 1 (3.70%) | 0.958 |
| Unknown | 30 (52.63%) | 15 (50.00%) | 15 (55.56%) | 0.878 |

Abbreviation: EGFR, epidermal growth factor receptor; PD1, programmed cell death; PD-L1, programmed deathligand 1; TKI, tyrosine kinase inhibitor; VEGF, vascular endothelial growth factor.

**Table S4.** Treatment failure patterns in various interventions

|  | All (n=57) | TKIs (n=30) | TKIs+uLT (n=27) | P |
| --- | --- | --- | --- | --- |
| LPD | 14 (24.56%) | 5 (16.67%) | 9 (33.33%) | 0.250 |
| IPD | 30 (52.63%) | 15 (50.00%) | 15 (55.56%) | 0.878 |
| EPD | 2 (3.51%) | 2 (6.67%) | 0 (0%) | 0.519 |
| Mixed PD | 11 (19.30%) | 8 (26.67%) | 3 (11.11%) | 0.250 |

Abbreviation: LPD, lung in situ progression; EPD, extracranial metastasis progression; IPD, intracranial progression.

**Table S5.** Treatment failure patterns in different number of BM lesions

|  | All (n=57) | Oligo-BM (n=26) | Extensive BM (n=31) | P |
| --- | --- | --- | --- | --- |
| LPD | 14 (24.56%) | 8 (30.77%) | 6 (19.35%) | 0.491 |
| IPD | 30 (52.63%) | 14 (53.85%) | 16 (51.61%) | 1.000 |
| EPD | 2 (3.51%) | 1 (3.85%) | 1 (3.23%) | 1.000 |
| Mixed PD | 11 (19.30%) | 3 (11.54%) | 8 (25.81%) | 0.307 |

Abbreviation: LPD, lung in situ progression; EPD, extracranial metastasis progression; IPD, intracranial progression.

**Table S6.** Tumor volume assessment in various interventions

|  | All (n=50) | TKIs (n=29) | TKIs+uLT (n=21) | P |
| --- | --- | --- | --- | --- |
| <0 | 2 (4.00%) | 1 (3.45%) | 1 (4.76%) | 0.921 |
| 0-25% | 2 (4.00%) | 1 (3.45%) | 1 (4.76%) |  |
| 25%-50% | 6 (12.00%) | 4 (13.79%) | 2 (9.52%) |  |
| 50%-75% | 7 (14.00%) | 6 (20.69%) | 1 (4.76%) |  |
| 75%-100% | 33 (66.00%) | 17 (58.62%) | 16 (76.19%) |  |

**Table S7.** Tumor volume assessment in different number of BM lesions

|  | All (n=50) | Oligo-BM (n=21) | Extensive BM (n=29) | P |
| --- | --- | --- | --- | --- |
| <0 | 2 (4.00%) | 0 (0%) | 1 (3.45%) | 0.152 |
| 0-25% | 2 (4.00%) | 1 (4.76%) | 1 (3.45%) |  |
| 25%-50% | 6 (12.00%) | 2 (9.52%) | 2 (6.90%) |  |
| 50%-75% | 7 (14.00%) | 4 (19.05%) | 1 (3.45%) |  |
| 75%-100% | 33 (66.00%) | 14 (66.67%) | 16 (55.17%) |  |


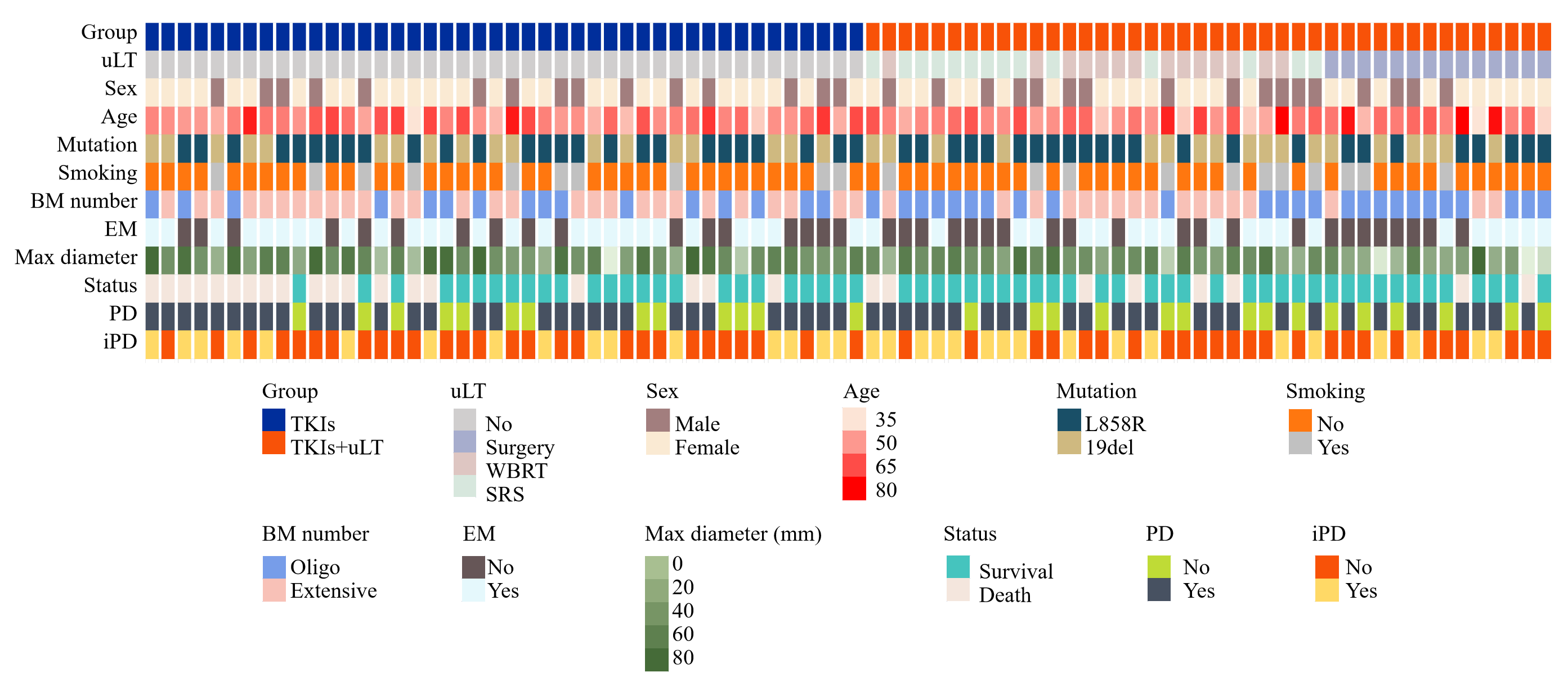


**Figure S1.** Characteristics of patients in two groups

Abbreviation: BM, brain metastasis; EM, extracranial metastasis; PD, progressive disease; iPD, intracranial progressive disease.

**
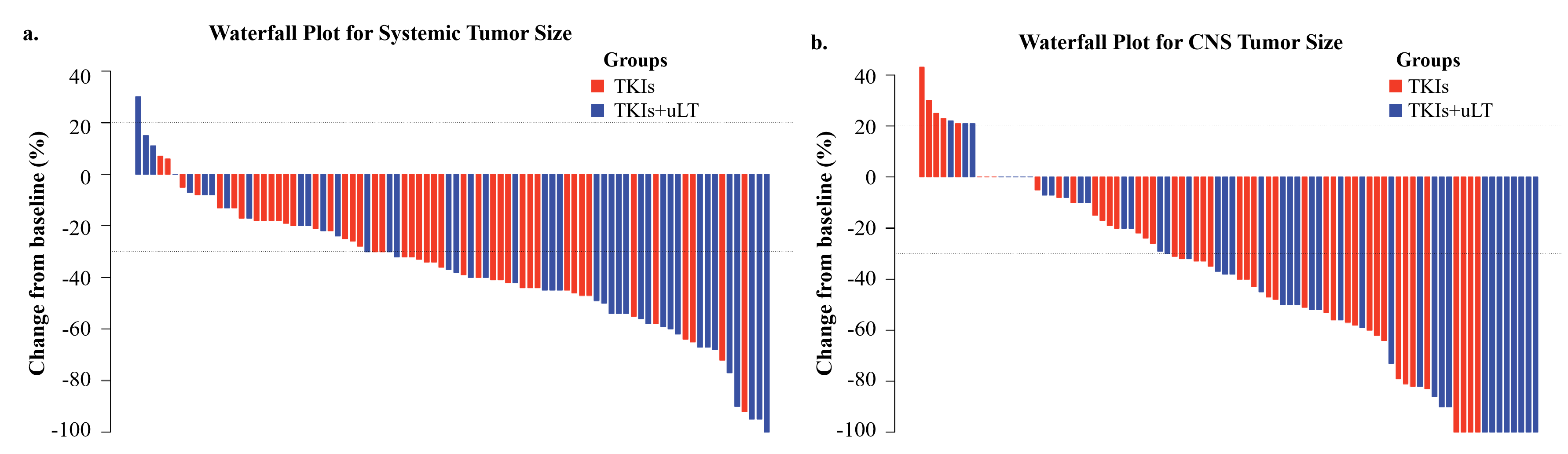
**

**Figure S2.** Waterfall plot for systemic tumor size (a) and CNS tumor size (b)

Abbreviation: CNS, central nervous system.


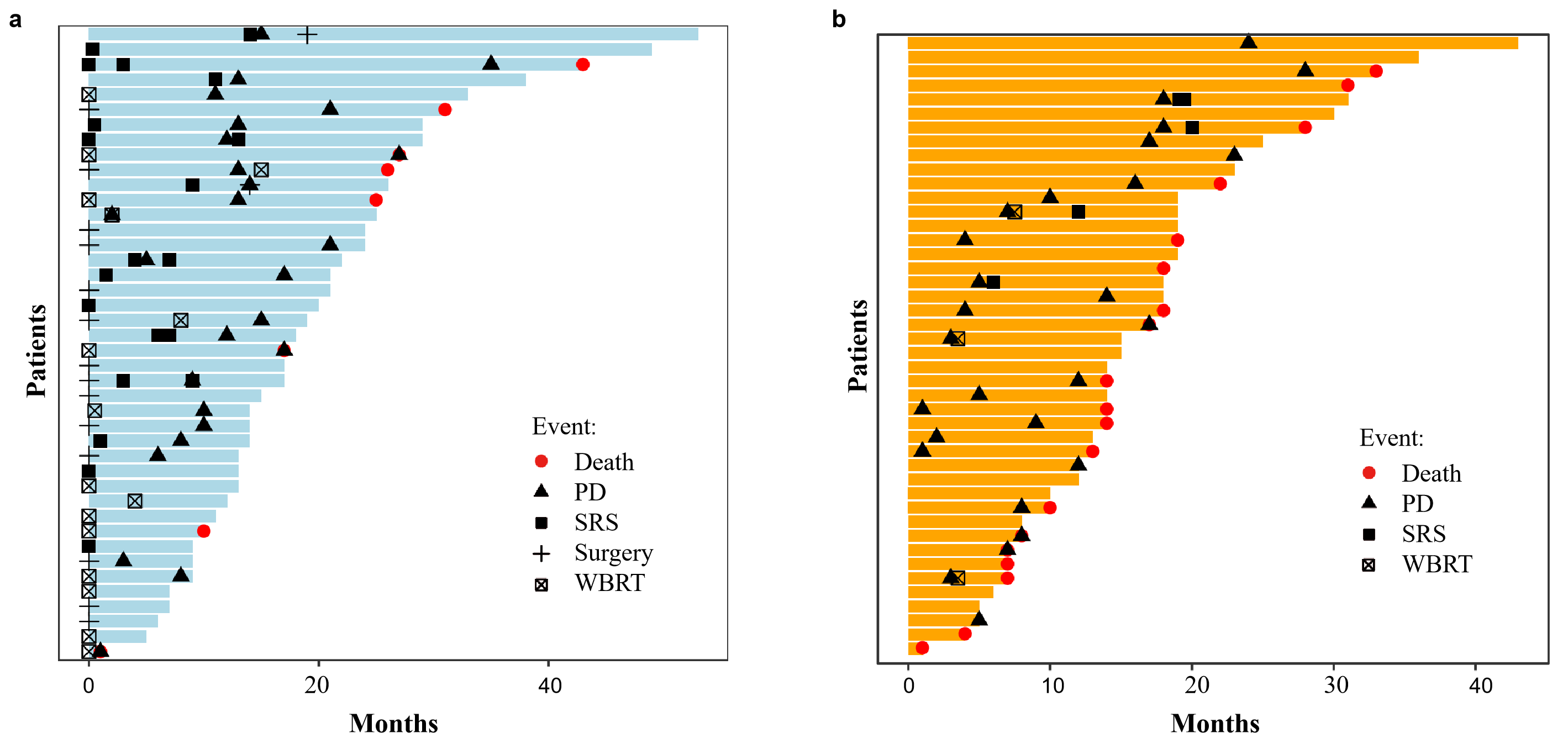


**Figure S3.** Swimmers plot of individual patients who were treated with TKIs (a) or TKIs+uLT (b)
